# Pulmonary scedosporiosis in an intractable immunocompetent host: A case report and literature review

**DOI:** 10.1111/crj.13739

**Published:** 2024-03-03

**Authors:** Hong Yu Liang, Chun Hua Han, Wen Juan Xu, Jian Sun, Qiang Wang

**Affiliations:** ^1^ Department of Respiratory and Critical Care Medicine The Affiliated Hospital of Qingdao University Qingdao China; ^2^ Department of Bacteriology Room The Affiliated Hospital of Qingdao University Qingdao China

**Keywords:** fungal infections, immunocompetent host, pulmonary scedosporiosis, scedosporiosis, *Scedosporium apiospermum*, treatment of scedosporiosis

## Abstract

Pulmonary scedosporiosis is a rare pulmonary infection that often presents with nonspecific symptoms and radiological findings. In this report, we present a case of localized pulmonary scedosporiosis in an immunocompetent patient and analyze a total of 25 immunocompetent patients with pulmonary scedosporiosis. Through this case and the literature, we highlight the importance of considering pulmonary scedosporiosis in patients with nonspecific clinical symptoms and radiological findings resembling aspergilloma. This case and the literature further emphasize the significance of surgical intervention. Regardless of the use of antifungal drugs, surgery should be conducted as soon as possible.

## Graphical abstract

We have reported a case and analyzed a total of 25 immunocompetent patients with the rare pulmonary scedosporiosis. Through analysis, we have reached the conclusion that early surgery is quite necessary. We hope our work is helpful for the diagnosis and treatment of this rare fungal infection.

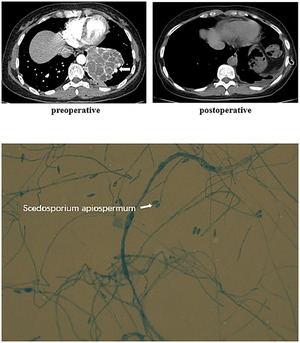

## 1 BACKGROUND

*Scedosporium apiospermum* as a member of the genus *Scedosporium* is a rare opportunistic fungus which are commonly isolated from sewage and rural soil. It always led to severe pulmonary invasive infection in immunocompromised host or patients suffering from severe trauma.^1^ However, in immunocompetent patients, pulmonary infections caused by *S. apiospermum* have been rarely reported and there is no uniform treatment standard. How to use antifungal drugs rationally and whether to operate as soon as possible are still controversial.^2^ Making an early diagnosis and optimal treatment judgment is crucial for the prognosis of this disease. Here, we report a case of an immunocompetent patient who suffered from a localized pulmonary infection caused by *S. apiospermum*. After diagnosis, we gave her 6 months of treatment with voriconazole. The symptoms and radiological imagine improved but suddenly recurred after the voriconazole withdrawal. Ultimately, she underwent surgical intervention and experienced favorable outcomes. At the same time, we reviewed 25 cases of lung infection caused by the *Scedosporium* spp with normal immune function and discussed their treatment and prognosis.

## 2 CASE PRESENTATION

**FIGURE 1:**
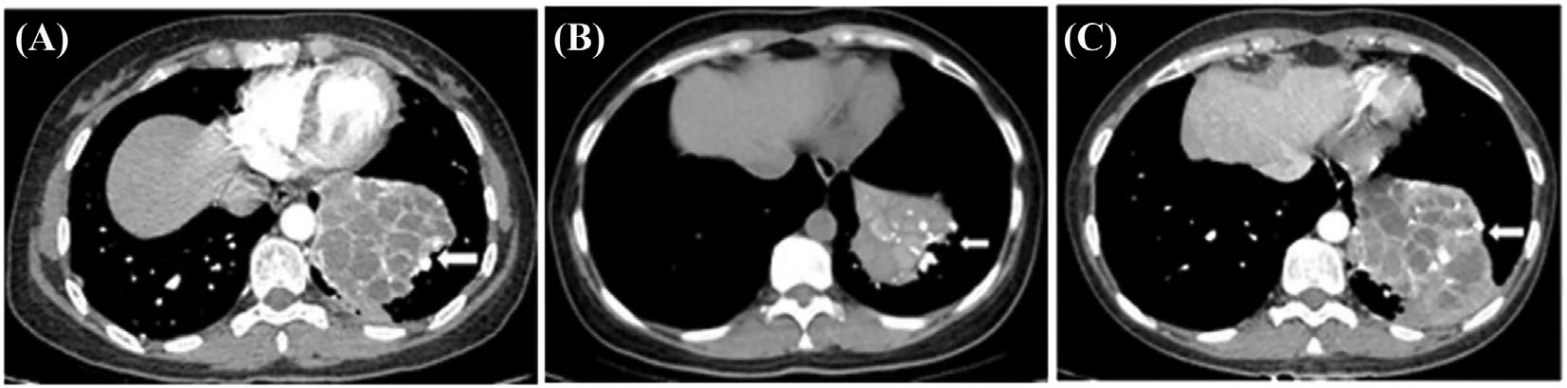
Computed tomography scan of chest, showing huge irregular solid mass with internal compartmentation in the lower lobe of the left lung: (A) before treatment with voriconazole, (B) after 6 months of treatment with voriconazole, and (C) after 3 weeks of discontinuation of treatment with voriconazole.

A 49‐year‐old woman came to our hospital with complaints of recurrent episodes of cough and hemoptysis for the last 5 years. She had a history of pulmonary tuberculosis 10 years before. Physical examination finding included moist rales in the left lower part of chest. CT revealed a huge irregular solid mass with internal compartmentation in the lower lobe of the left lung (Figure 1A). There was no significant increase in peripheral blood leukocyte count and CRP. Then, we irrigated the bronchus and biopsied the lesion site many times via fiberoptic bronchoscope. To make a clear diagnosis, we also did a percutaneous lung biopsy. All specimens obtained were sent for culture and histopathology. However, neither *Mycobacterium tuberculosis* nor nonspecific bacteria grew from any of the bronchial washing and lung puncture specimens, and there was no evidence of fungal infection found either by direct microscopic observation or by PAS and GMS staining. Finally, the patient was discharged with a diagnosis of old pulmonary tuberculosis.

**FIGURE 2:**
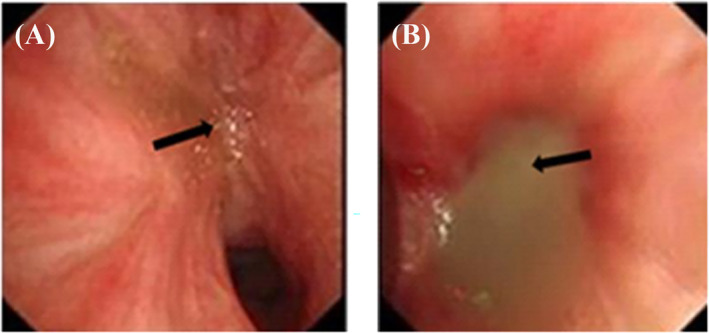
Scan of the diseased site under fiberoptic bronchoscopy: (A) Left inferior lobar bronchus was narrow and nearly occluded, and (B) during cleaning up of the diseased site, a large amount of yellowish white pus flowed out of the left inferior lobar bronchus.

**FIGURE 3:**
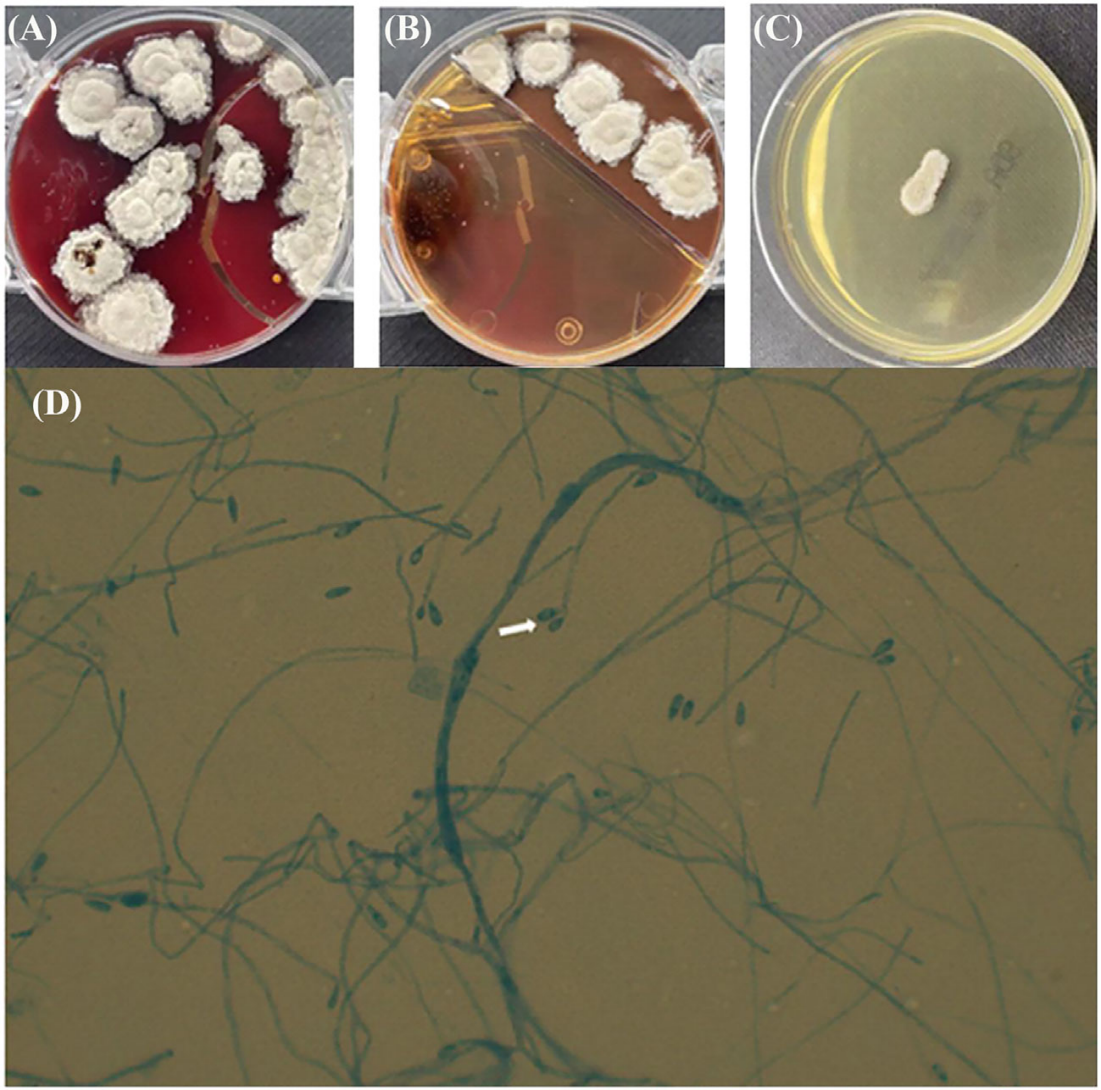
The white, goat hair like *Scedosporium apiospermum* colonies on (A) chocolate agar plate, (B) Sabouraud's agar medium, and (C) Columbia blood base and (D) magnification ×40, lactophenol cotton blue prepared slide from culture showing oval conidia with scar at the base, larger end toward the apex.

**FIGURE 4:**
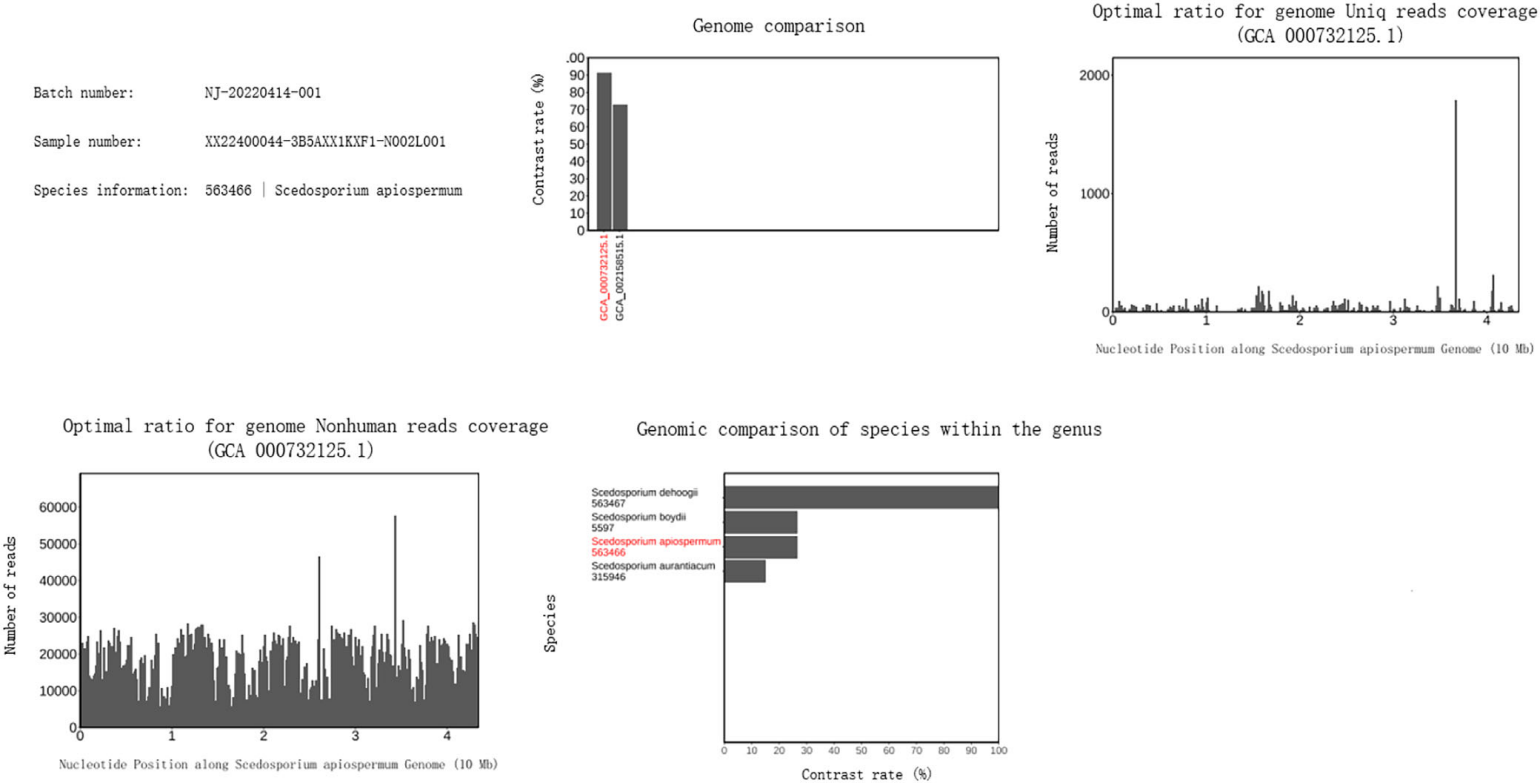
Results of gene sequencing of sputum samples indicate *Scedosporium apiospermum*.

**FIGURE 5:**
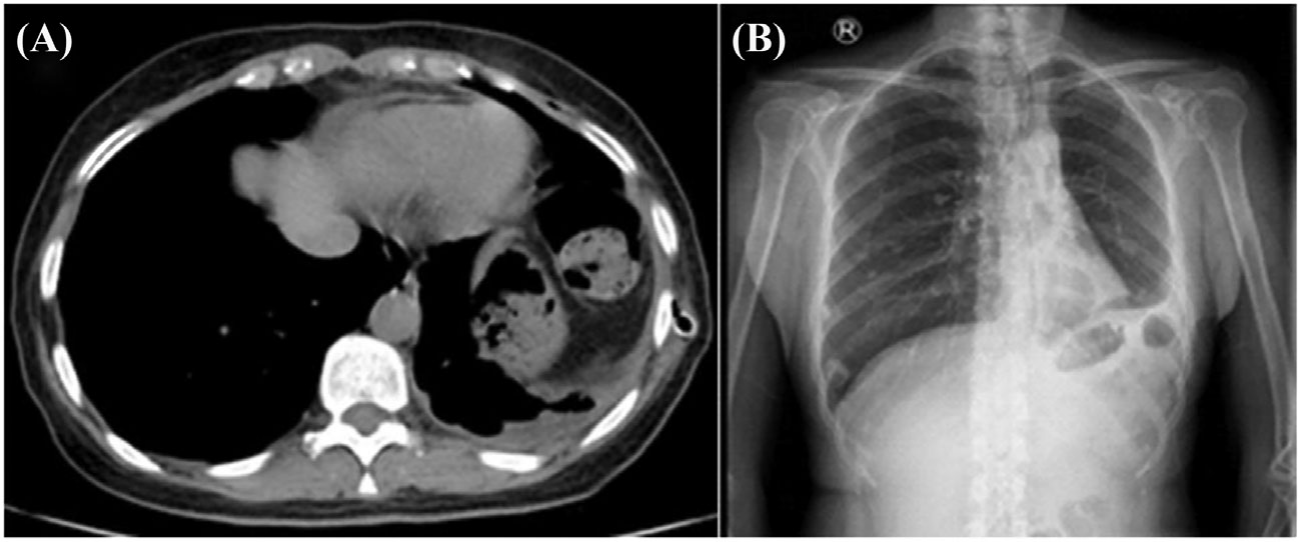
(A) CT and (B) chest X‐ray images of the patient undergoing left lower lobectomy.

After being discharged, the patient still coughed and had more frequent hemoptysis. Finally, she returned to our hospital 2 years later. We irrigated the bronchus again and sent the bronchoalveolar lavage fluid to culture and the gene sequencing. Under the bronchoscopy image, we found the lumens of the dorsal and basal segments of the left lower lobe were narrow and nearly occluded (Figure 2A). During cleaning up of the diseased site, a large amount of yellowish white pus flowed out of them (Figure 2B). The lavage culture remained negative, but sputum culture after incubation on Columbia blood base (Figure 3A) and chocolate agar plate (Figure 3B) both yielded white, goat hair like fungal colonies. The fungal colonies went down to posterity of Sabouraud's agar medium (Figure 3C) and carried out lactic acid carbolic cotton blue stain solution dyeing (Figure 3D), which were identified as *S. apiospermum* under the microscope. Results of gene sequencing of sputum samples confirmed it again (Figure 4). After diagnosis, therapy with oral voriconazole (200 mg/12 h) was started. After 6 months of treatment with antifungal medicine, the chest CT showed the improvement of the left lower lobe mass (Figure 1B) and we stopped the voriconazole. After 3 weeks, the patient had a sudden fever and the chest CT showed progression disease than before (Figure 1C). We restarted voriconazole and proceeded with a left lower lobectomy after her condition stabilized. Following the surgical intervention, the patient experienced substantial improvement in symptoms and imaging results (Figure 5). A follow‐up period of over 5 months revealed a notable reduction in chest tightness and suffocation as reported by the patient, along with the absence of persistent cough or phlegm.

## 3 LITERATURE REVIEW

*S. apiospermum* is a rare opportunistic fungus, which always infects immunocompromised hosts or patients suffering from severe trauma. It is very rare for this fungus to infect immunocompetent hosts, and there is a lack of corresponding diagnosis and treatment guidelines.^1^ We searched “PubMed” and “Web of Science” with the search strategy (“Scedosporiosis” or “Scedosporium” or “Pseudallescheria boydii” and “immunocompetent” and “lung”) and organized the published cases of pulmonary scedosporiosis in an immunocompetent host. We excluded patients suffering from severe trauma such as drowning, serious trauma, and lung transplantation. Twenty‐five cases including the current case were identified and analyzed (Table 1) in order to find out some regular results and provide reference for clinical treatment of these patients. For more details and analysis of the twenty‐five cases mentioned above, please consult the supporting information (Tables S1–S6 and Data S1).

**TABLE 1:** Clinical characteristics, cardinal symptoms, CT/chest radiography, diagnosis, and treatment/outcome of 25 cases of immunocompetent patients with pulmonary scedosporiosis.

Cases	Age/sex	Associated lung diseases/risk factors	Cardinal symptoms	CT/chest radiography	Diagnosis	Treatment/outcome	Years/reference
1	65/M	TB	Cough, bloody sputum, dyspnea, and weight loss	Aspergilloma with cavity, infiltrates, and atelectasis	Postmortem pathology	Anti‐TB/death	1982/^3^
2	69/M	None	Cough, bloody sputum, and chest pain	Aspergilloma with cavity and fibrotic infiltrates	Spinal tissue culture	Amphotericin B/lost	1994/^4^
3	54/F	TB	Cough, dyspnea, fever, and weight loss	Micronodular infiltrates	BALF culture	Ketoconazole/cured	1997/^5^
4	74/F	None	Cough	Intrabronchial lump	TBLB culture	Itraconazole/cured	1997/^6^
5	41/F	TB	Cough, purulent sputum, and fever	Aspergilloma with cavity	Sputum culture	Ketoconazole, GC/death	1998/^7^
6	61/F	Emphysema and hypertension	Cough, hemoptysis, fever, night sweats, and weight loss	Aspergilloma with cavity	BALF culture	Amphotericin B/death	1999/^8^
7	58/F	None	Cough	Atelectasis	TBLB culture	Surgery/cured	2001/^9^
8	72/F	COPD, lymphoma, and GC	Cough and fever	Infiltrates	BALF and TBLB culture	Itraconazole/death	2003/^10^
9	32/M	TB	Cough, hemoptysis, fever, and chest pain	Infiltrates	BALF culture	Miconazole/cured	2004/^11^
10	45/M	Rheumatoid arthritis and GC	Cough and hemoptysis	Aspergilloma with cavity	Postoperative pathology	Surgery/cured	2004/^11^
11	36/F	Diabetes	Cough, purulent sputum, dyspnea, fever, and chest pain	Aspergilloma with cavity	Postoperative pathology	Surgery/cured	2004/^11^
12	57/M	TB	Cough, hemoptysis, and chest pain	Aspergilloma with cavity and atelectasis	Postoperative pathology	Surgery/cured	2004/^12^
13	68/M	TB	Cough, hemoptysis, dyspnea, fever, asthenia, anorexia, night sweats, and weight loss	Aspergilloma with cavity and consolidation	Sputum culture	Voriconazole/death	2007/^13^
14	42/M	TB	Cough and hemoptysis	Aspergilloma with cavity	BALF culture	Surgery/cured	2010/^14^
15	27/M	TB	Cough, hemoptysis, and fever	Aspergilloma with cavity, consolidation, and bronchiectasis	Postoperative pathology	Surgery/cured	2010/^15^
16	71/M	None	Cough, purulent sputum, and fever	Aspergilloma with cavity	BALF and lung puncture tissue culture	Voriconazole/cured	2011/^16^
17	61/F	TB	Cough, hemoptysis, fever, and weight loss	Aspergilloma with cavity, consolidation, and bronchiectasis	BALF and sputum culture	Voriconazole/cured	2011/^17^
18	53/M	None	Cough, fever, maculopapular, and jaundice	Diffuse infiltrates and bilateral pleural effusion	Postmortem pathology	Amphotericin B/death	2012/^18^
19	47/M	TB	Cough, hemoptysis, purulent sputum, and dyspnea	Aspergilloma	Sputum culture	Surgery/cured	2014/^19^
20	40/M	TB	Cough and hemoptysis	Aspergilloma with cavity	Lung puncture tissue culture	Voriconazole/cured	2016/^20^
21	51/F	None	Cough and night sweats	Aspergilloma with cavity and bronchiectasis	TBLB culture	Surgery/lost	2017/^21^
22	73/F	Hypertension	None	Aspergilloma	BALF and TBLB culture and gene sequencing	Surgery/cured	2018/^22^
23	44/F	None	Cough, hemoptysis, bloody sputum, night sweats, and weight loss	Aspergilloma with cavity and consolidation	BALF culture	Surgery/cured	2020/^23^
24	83/F	COPD, hypertension, and atrial fibrillation	Cough, bloody sputum, and dyspnea	Bronchiectasis	BALF and sputum culture	Voriconazole/cured	2021/^24^
25	49/F	TB	Cough, hemoptysis, and purulent sputum	Aspergilloma	Sputum culture and gene sequencing	Surgery/cured	2023/present case

The clinical symptoms of pulmonary scedosporiosis are nonspecific. Patients can present with cough, sputum, and blood in the sputum. When the focal encroach bronchial arteries, patients may have hemoptysis. As the focal expands and compresses or blocks a larger bronchus, patients may present with wheezing and dyspnea. In addition, many patients also have constitutional symptoms, among which fever, night sweats, and weight loss are more common. Because pulmonary scedosporiosis often occurs in patients with underlying lung diseases, such as tuberculosis, pulmonary cystic fibrosis, and bronchiectasis, it is often mistaken for the preexisting diseases causing the clinical symptoms.^25^, ^26^ It is also difficult to diagnose pulmonary scedosporiosis from radiological imagines, because its imaging findings are very similar to pulmonary aspergillosis. The focal may present as aspergilloma with or without cavity which is similar to aspergillus tumor. For immunocompetent patients, *S. apiospermum* always leads to localized lung infection. However, there are also a small number of cases mainly manifested by pulmonary inflammatory infiltration, which can involve single lung lobe or bilateral multiple lung lobes.^1^, ^24^ For nonspecific clinical symptoms and indistinguishable radiological imagines, pulmonary infections caused by rare fungi should be considered.

Obtaining suitable samples and culturing or conducting pathological test on them is important to diagnose pulmonary scedosporiosis. In this study, sputum as the easiest available specimen accounted for only one fourth of the specimens for definitive diagnosis but BALF and TBLB accounted for 62.5%. In addition, two patients were diagnosed by percutaneous lung biopsy, and one patient was diagnosed by spinal tissue culture invaded by *S. apiospermum*. It is notable that *S. apiospermum* and *Aspergillus* have similar microscopic structure. It is always difficult to distinguish them by direct microscopy.^1^ In recent years, clinical application of pathogen gene sequencing has been widely developed. The technology provides a strong support for the early diagnosis of rare pathogen infection due to its advantages such as high sensitivity, no need for culture, and fast detection speed.^27^ We believe that taking multiple samples and sending them for culturing or pathology in combination with pathogen genetic sequencing may be a better option for diagnosing the disease as quickly as possible.

At present, there is no uniform standard of treatment for pulmonary scedosporiosis. According to drug sensitive test to anti‐*S. apiospermum* mentioned in documents, voriconazole is currently considered to be the most sensitive drug for the treatment of *S. apiospermum*. Besides, voriconazole can go through the blood–brain barrier and plays a role in the central nervous system. For patients with definite diagnosis, voriconazole should be used as soon as possible, regardless of whether they have immune deficiency.^28^ However, our study found that only 4 of the 10 patients who received voriconazole showed significant improvement. The efficacy of other antifungal drugs is also poorly effective, and the overall effective rate of antifungal drugs is only one third.

Surgery has shown good results, with 10 of the 12 patients who underwent surgery, regardless of whether they had been treated with antifungal drugs, showing significant clinical improvement after surgery. There is literature pointing out that, in addition to antifungal treatment, lesion resection should be performed if the patient's condition permits, regardless of whether the patient has compromised immune function.^29^ This result ties well with our study.

Some localized lesions will progress to invasive pulmonary fungal infection, which will lead to a very poor prognosis. These patients often die of respiratory failure due to severe and diffuse fungal infection of lung, but the infected lesions often require thorough surgical debridement to cure.^1^

## 4 CONCLUSION

The clinical symptoms and radiological imagines of pulmonary infection caused by *S. apiospermum* in immunocompetent people are nonspecific, and it is difficult to make a clear diagnosis. Repeated and multiple samples culturing and pathology combined with pathogen gene sequencing is necessary for early diagnosis. In terms of treatment, the prognosis of pulmonary scedosporiosis is always poor. Early surgery should be conducted if the patient's condition permits, whether antifungal drugs are used or not.

## AUTHOR CONTRIBUTIONS

Hongyu Liang and Qiang Wang wrote the main manuscript text, and Chunhua Han identified *Scedosporium apiospermum* in the laboratory and prepared Figure 3. Wenjuan Xu and Jian Sun perform multiple fiberoptic bronchoscopy examinations for the patient and prepared Figure 2. All authors contributed to the writing of the final manuscript and reviewed the final manuscript.

## CONFLICT OF INTEREST STATEMENT

There are no potential conflicts of interest of this article.

## ETHICS STATEMENT

Appropriate written informed consent was obtained from the patient for the publication of this case report and accompanying images. It was approved by the Clinical Research Ethics Committee of The Affiliated Hospital of Qingdao University and was implemented.

## Supporting information


**Table S1.** References and year of their publication.
**Table S2.** Review of treatment and outcome in immunocompetent patients with pulmonary scedosporiosis.
**Table S3.** Patient characteristics of 25 immunocompetent pulmonary scedosporiosis patients.
**Table S4.** Cardinal symptoms of 25 immunocompetent pulmonary scedosporiosis patients.
**Table S5.** CT/Chest radiography of 25 immunocompetent pulmonary scedosporiosis patients.
**Table S6.** Treatment/Outcome of 25 immunocompetent pulmonary scedosporiosis patients.

**Data S1.** Supporting Information.

## Data Availability

All the relevant data and material are available from the corresponding author on reasonable request.
