# Supplementary material for: Pulmonary scedosporiosis in an intractable immunocompetent host: A case report and literature review
Source: Clin Respir J. 2024 Mar 3;18(3):e13739. doi: 10.1111/crj.13739 (PMC10909920; doi:10.1111/crj.13739)
Supplement: Supplementary file 1 — Table S1. References and year of their publication. Table S2. Review of treatment and outcome in immunocompetent patients with pulmonary scedosporiosis. Table S3. Patient characteristics of 25 immunocompetent pulmonary scedosporiosis patients. Table S4. Cardinal symptoms of 25 immunocompetent pulmonary scedosporiosis patients. Table S5. CT/Chest radiography of 25 immunocompetent pulmonary scedosporiosis patients. Table S6. Treatment/Outcome of 25 immunocompetent pulmonary scedosporiosis patients. [file CRJ-18-e13739-s002.docx]

**Supplemental Table 1. References and year of their publication**

| Cases | Year | References |
| --- | --- | --- |
| 1 | 1982 | Petriellidium boydii fungus ball in a patient with active tuberculosis |
| 2 | 1994 | Invasive pulmonary pseudallescheriasis with direct invasion of the thoracic spine in an immunocompetent patient |
| 3 | 1997 | Pulmonary tuberculosis associated with invasive pseudallescheriasis |
| 4 | 1997 | Intrabronchial Pseudallescheriasis in an Immunocompetent Woman |
| 5 | 1998 | Pulmonary scedosporiosis |
| 6 | 1999 | Disseminated Pseudallescheria boydii infection in a nonimmunocompromised host |
| 7 | 2001 | Pulmonary infection with Scedosporium prolificans in an immunocompetent individual |
| 8 | 2003 | Fatal pulmonary scedosporiosis |
| 9 | 2004 | Respiratory tract intracavitary colonization due to Scedosporium apiospermum: report of four cases |
| 10 | 2004 | Respiratory tract intracavitary colonization due to Scedosporium apiospermum: report of four cases |
| 11 | 2004 | Respiratory tract intracavitary colonization due to Scedosporium apiospermum: report of four cases |
| 12 | 2004 | Pulmonary pseudallescheriasis in a patient with healed tuberculosis |
| 13 | 2007 | Scedosporium apiospermum lung infection with fatal subsequent postoperative outcome in an immunocompetent host |
| 14 | 2010 | Scedosporium apiospermum presenting as a pulmonary mycetoma in immunocompetent male |
| 15 | 2010 | Pseudallescheria boydii Lung Infection in an Immunocompetent Adult, Difficulties in Diagnosis and Management |
| 16 | 2011 | [A case of lung scedosporiosis successfully treated with monitoring of plasma voriconazole concentration level] |
| 17 | 2011 | A 61 year-old female with a prior history of tuberculosis presenting with hemoptysis |
| 18 | 2012 | Dapsone hypersensitivity syndrome complicated by Scedosporium apiospermum pneumonia in an immunocompetent patient |
| 19 | 2014 | Invasive lung infection by Scedosporium apiospermum in an immunocompetent individual |
| 20 | 2016 | Pulmonary scedosporiosis mimicking aspergilloma in an immunocompetent host: a case report and review of the literature |
| 21 | 2017 | Scedosporium apiospermum Mycetoma in an Immunocompetent Patient without Prior Lung Disease |
| 22 | 2018 | Pulmonary Scedosporium apiospermum Infection with Pulmonary Tumorlet in an Immunocompetent Patient |
| 23 | 2020 | Management of pulmonary Scedosporium apiospermum infection by thoracoscopic surgery in an immunocompetent woman |
| 24 | 2021 | Scedosporium apiospermum Pneumonia in an Immunocompetent Host |
| 25 | 2023 | This article |

**Supplemental Table 2. Review of** **treatment and outcome in immunocompetent patients with pulmonary scedosporiosis**

| Cases | Age/Sex | Treatment | Outcome |
| --- | --- | --- | --- |
| 1 | 65/M | Empirical anti-tuberculosis treatment | Death (sudden death four days later) |
| 2 | 69/M | Amphotericin B | Lost |
| 3 | 54/F | Miconazole nitrate 600Mg/D for 10 weeks (no improvement), change to ketoconazole 300Mg/D for 1 year and anti-tuberculosis | Clinical improvement (follow up for 2 years) |
| 4 | 74/F | Itraconazole 100Mg/D for 12 weeks  (no improvement), change to itraconazole 200Mg/D | Clinical improvement |
| 5 | 41/F | Ketoconazole 400Mg/D and prednisone 20Mg/D for 1 year | Death (death due to worsening symptoms after 1 year) |
| 6 | 61.F | Amphotericin B 1000Mg/D for several months | Death (death due to diffuse fungal pneumonia and pulmonary embolism after several months) |
| 7 | 58/F | Surgery | Clinical improvement (no symptoms within 6 months of follow-up) |
| 8 | 72/F | Itraconazole | Death (death due to Septic shock) |
| 9 | 32/M | Miconazole 400mg/d | Clinical improvement (follow up for 1 month) |
| 10 | 45/M | Surgery | No recurrence in 2 years |
| 11 | 36/F | Surgery | No recurrence |
| 12 | 57/M | Surgery | No recurrence in 1 year |
| 13 | 68/M | Oral voriconazole for 1 month (no improvement),  Surgery | Death (death due to respiratory failure caused by severe pneumonia) |
| 14 | 42/M | Surgery | Clinical improvement |
| 15 | 27/M | Multiple antifungal drugs (itraconazole, voriconazole, amphotericin B, no improvement), surgery | Clinical improvement |
| 16 | 71/M | Voriconazole 200Mg/D for 2 months  (no improvement), change to voriconazole 400Mg/D | Clinical and radiological improvement |
| 17 | 61/F | Voriconazole | Clinical improvement |
| 18 | 53/M | Amphotericin B | Death (death due to respiratory failure) |
| 19 | 47/M | Voriconazole for 4 weeks, surgery | No recurrence |
| 20 | 40/M | Voriconazole 800Mg/D for the first day and 400Mg/D for 4 months | Radiological improvement |
| 21 | 51/F | Voriconazole 400Mg/D for 3 months  (no improvement), surgery | Lost |
| 22 | 73/F | Surgery | No recurrence in 5 years |
| 23 | 44/F | Voriconazole 400Mg/D for 2 months  (no improvement), surgery | No recurrence in 1.5 years |
| 24 | 83/F | Voriconazole 400Mg/D | Clinical improvement |
| 25 | 49/F | Voriconazole 400Mg/D for 6 months (no improvement), surgery | Clinical and radiological improvement |

**Supplemental Table 3. Patient characteristics of 25 immunocompetent pulmonary scedosporiosis patients**

| Characteristics | Number (%) or median (IQR) |
| --- | --- |
| Age, years | 54 (43-68.5) |
| Sex, male | 12 (48%) |
| Underlying lung disease | 15（60%） |
| Pulmonary tuberculosis | 12（48%） |
| Chronic obstructive pulmonary disease | 2（8%） |
| Emphysema | 1（4%） |
| Extrapulmonary basic diseases or risk factors | 6（24%） |
| Hypertension | 3（12%） |
| Long-term glucocorticoid use | 2（8%） |
| Diabetes | 1（4%） |
| Lymphoma | 1（4%） |
| Rheumatoid arthritis | 1（4%） |
| Atrial fibrillation | 1（4%） |

*IQR, interquartile range.

**Supplemental Table 4. Cardinal symptoms of 25 immunocompetent pulmonary scedosporiosis patients**

| Cardinal symptoms | Number (%) |
| --- | --- |
| Pulmonary symptoms | 24 (96%) |
| Cough | 24 (96%) |
| Hemoptysis | 12 (48%) |
| Dyspnea | 6 (24%) |
| Purulent sputum | 5 (20%) |
| Bloody sputum | 4 (16%) |
| Chest pain | 4 (16%) |
| Constitutional symptom | 14 (56%) |
| Fever | 11 (44%) |
| Weight loss | 6 (24%) |
| Night sweats | 4 (16%) |
| Asthenia | 1 (4%) |
| Anorexia | 1 (4%) |
| Maculopapular | 1 (4%) |
| Jaundice | 1 (4%) |

**Supplemental Table 5. CT/Chest radiography of 25 immunocompetent pulmonary scedosporiosis patients**

| CT/Chest radiography | Number (%) |
| --- | --- |
| Aspergilloma with or without cavity | 18 (72%) |
| Aspergilloma with cavity | 15 (60%) |
| Aspergilloma without cavity | 3 (12%) |
| Infiltrates^*^ | 6 (24%) |
| Consolidation | 4 (16%) |
| Bronchiectasis | 4 (16%) |
| Atelectasis | 3 (12%) |
| Intrabronchial lump | 1 (4%) |
| Bilateral pleural effusion | 1 (4%) |

*Infiltration types specially pointed out in the documents, fibrotic infiltrates（n=1）, micronodular infiltrates (n=1), diffuse infiltrates (n=1), not specially pointed out in the documents (n=3).

**Supplemental Table 6. Treatment/Outcome of 25 immunocompetent pulmonary scedosporiosis patients**

| Treatment(number) | | Outcome(number) | | |
| --- | --- | --- | --- | --- |
|  |  | Improvement/  No recurrence | No improvement  /Death | Lost/Not mentioned |
| Voriconazole | 10 | 4 | 5 | 1 |
| Amphotericin B | 4 | 0 | 3 | 1 |
| Itraconazole | 3 | 1 | 2 | 0 |
| Miconazole | 2 | 1 | 1 | 0 |
| Ketoconazole | 2 | 1 | 1 | 0 |
| Surgery | 12 | 10 | 1 | 1 |
