# Supplementary material for: Pulmonary scedosporiosis in an intractable immunocompetent host: A case report and literature review
Source: Clin Respir J. 2024 Mar 3;18(3):e13739. doi: 10.1111/crj.13739 (PMC10909920; doi:10.1111/crj.13739)
Supplement: Supplementary file 2 — Data S1. Supporting Information. [file CRJ-18-e13739-s001.docx]

**Literature review**

**Patients and methods**

**Patients**

We searched “PubMed” and ”Web of Science” with the search strategy (("Scedosporiosis “or "Scedosporium" or"Pseudallescheria boydii “) and “immunocompetent” and” lung”) and organized the published cases of pulmonary scedosporiosis in an immunocompetent host. We excluded patients suffering from severe trauma such as drowning, serious trauma, lung transplantation, etc. 24 cases were identified on “PubMed” and ”Web of Science”. Thus including current case, a total of 25 cases were analyzed (Supplemental Table 1).

**Date collection**

We collected the following data in these cases. patient characteristics (age, sex, associated lung diseases/risk factors), cardinal symptoms, CT/chest radiography, diagnosis, treatment/outcome (Table 1), and analyzed this information. To facilitate analysis, the specific treatment and outcome of all patients are compiled into a separate table (Supplemental Table 2).

**Results**

**Patient characteristics**

A total of 25 (n=25) patients were included in this study. The median age of patients was 54 years (interquartile range (IQR) 43-68.5 years), the maximum age was 83 years, and the minimum age was 27 years, including 12 (48%) men and 13 (52%) women.

18 (72%) patients had at least one associated lung disease or risk factor, of which 15 (60%) patients had underlying lung diseases. Among the underlying lung diseases, TB accounted for the highest proportion, with 12 (48%) patients. In addition, 2 patients had COPD and 1 patient had emphysema. Six (24%) patients had extrapulmonary basic diseases or risk factors, including hypertension (n=3), long-term glucocorticoid use (n=2), diabetes (n=1), lymphoma (n=1), rheumatoid arthritis (n=1), and atrial fibrosis (n=1) (Supplemental Table 3).

**Cardinal symptoms**

Of the 25 patients, 24 (96%) had at least one pulmonary symptom, of which cough accounted for the highest proportion (n=24, 96%), followed by hemoptysis (n=12, 48%), dyspnea (n=6, 24%), purulent sputum (n=5, 20%), bloody sputum (n=4, 16%), chest pain (n=4, 16%). Fourteen (56%) patients had at least one constitutional symptom. The highest frequency of occurrence was fever (n=11,44%), followed by weight loss (n=6,24%), night sweats (n=4,16%), asthenia (n=1,4%), anorexia (n=1,4%), maculopapular (n=1,4%), and jaundice (n=1,4%) (Supplemental Table 4).

**CT/Chest radiography**

The results of all patients’ pulmonary CT/Chest radiography image had showed different degrees of pathological changes. Among the abnormal images, the most frequent occurrence was aspergilloma with or without cavity (n=18, 72%) and most of them have cavities (n=15, 60%), followed by Infiltrates (n=6, 24%), consolidation (n=4, 16%), bronchiectasis (n=4, 16%), atelectasis (n=3, 12%), intrabronchial lump (n=1, 4%), bilateral pleural effusion (n=1, 4%) (Supplemental Table 5).

**Diagnosis**

All patients were diagnosed by culture or pathology, 5 (8, 16, 17, 22, 24) patients were diagnosed by multiple culture methods, and 2 (22, 25) patients were diagnosed by gene sequencing technology at the same time. Of the 24 (n=24) culture samples, the samples from BALF accounted for 41.67% (n=10), the samples from sputum accounted for 25.00% (n=6), the samples from TBLB accounted for 20.83% (n=5), the samples from lung junction tissues accounted for 8.33% (n=2), and the samples from spinal tissues accounted for 4.17% (n=1). In addition, 4 (10,11,12,15) patients were diagnosed by postoperative pathology, and 2 (1,18) patients were not diagnosed before death, and they were diagnosed by postmortem pathology.

**Treatment and Outcome**

6 (24%) patients had not diagnosed before treatment. Among these 6 (1,10,11,12,15,18) patients, 2 (1,18) died. Patient 1 was misdiagnosed as tuberculosis and treated with anti-tuberculosis drugs. Four days later, the patient died suddenly. Patient 18 empirically applied the antifungal drug amphotericin B, but the curative effect was not ideal. The patient's condition deteriorated rapidly and eventually died of respiratory failure. The other 4 patients (10, 11, 12, 15) all had operations and achieved good prognoses. Among them, patient 15 was empirically treated with a variety of antifungal drugs (itraconazole, voriconazole, amphotericin B) before operation, but the effect was not good. Then he was treated with surgery, and the clinical symptoms were significantly improved after that.

19 (76%) patients had been diagnosed before treatment, of which 10 (40%) patients were treated with antifungal drugs alone. Of these 10 patients, 6 patients had clinical symptoms/images improved, 3 patients died, and 1 patient lost follow-up. 6 (24%) patients underwent surgical treatment after applying antifungal drugs. Of these 6 patients, 4 had a good prognosis, 1 died, and 1 lost follow-up. Of these 6 patients, it is noteworthy that 5 were mentioned with poor effect of antifungal drugs. Besides, 3 (12%) patients were treated with surgery alone and achieved good prognosis.

We analyzed all the patients who had used antifungal drugs, including the patients who had used antifungal drugs alone and the patients who had been treated with antifungal drugs combined with surgery. A total of 18 people were analyzed with their antifungal drugs and prognoses. Among them, 10 people had used voriconazole, of whom 4 had clinical and radiological improvement, 5 had no improvement, and 1 had not mentioned explicitly. There were 4 people who had used amphotericin B, of whom 1 had no improvement, 2 died and 1 lost follow-up. There were 3 people who had used itraconazole, of which 1 had clinical improvement, 1 had no improvement, and 1 died. 2 people had used miconazole, of whom 1 had clinical improvement, and another had not. 2 people had used ketoconazole, whom 1 had clinical improvement and one died. Among the 10 cases that had been treated with voriconazole, 6 cases clearly mentioned the therapeutic dose, all of which were 400Mg/D. It is notable that patient 16 had no obvious improvement after being treated with Voriconazole 200Mg/D for 2 months, but his clinical symptoms and radiological images were significantly improved after being treated with Voriconazole 400Mg/D.

We analyzed all patients who had been treated with surgery, including those who had been treated with surgery alone and those who had been treated with surgery after using antifungal drugs. A total of 12 people were analyzed with their prognoses. Among them, 10 patients had attained a favorable prognosis, 1 patient died and 1 patient lost follow-up (Supplemental Table 6).

Of the 25 patients, 6 died finally, with a mortality rate of 24%. There were 5 patients’ causes of death had been clearly described in cases. Respiratory failure caused by severe diffuse pulmonary fungal infection was the main cause of death (5, 13. 18). Other causes of death included septic shock (8) and pulmonary fungal embolism (6).
